# A Simulated Scenario to Improve Resident Efficiency in an Emergency Department

**DOI:** 10.7759/cureus.20462

**Published:** 2021-12-16

**Authors:** Ayanna Walker, Nubaha Elahi, Mary C Slome, Tracy MacIntosh, Maria Tassone, Latha Ganti

**Affiliations:** 1 Emergency Medicine, University of Central Florida College of Medicine, Orlando, USA

**Keywords:** multi-tasking, resident simulation, emergency medicine training, task shifting, graduate medical education (gme)

## Abstract

Introduction

Multitasking is a core competency in emergency medicine. Simulation has been shown to be an effective method of education, which allows learners to prepare for real-world challenges in a controlled environment.

Methods

In this study, trainees were given a scenario that simulated the experience of managing two patient encounters within a time metric while addressing interruptions that take place in a typical ED. Residents were evaluated using an internally developed scoresheet, which assessed task-switching abilities, documentation skills, and adherence to door to disposition time metric. Residents were asked to evaluate their experience with a survey.

Results

All the participants reported that they would translate some of the skills learned to their daily clinical practice. Five out of six residents reported improvements in their skills as a result of the task-switching training. The following three common themes were pervasive in the debrief discussion: (1) the residents felt the added pressure of the door-to-disposition metric, (2) the objectives of the simulation did not fit within their pre-constructed concept of a successful simulation equating to establishing the correct diagnosis, and (3) the interruptions were very realistic.

Discussion

Emergency physicians are interrupted approximately every 9-14 minutes, and this number increases with the number of patients being managed simultaneously. By developing a safe, simulated training environment, we sought to transfer key strategies for improving focus and learning to prioritize while also helping them to identify how certain pressures and interruptions affected their stress levels and concentration.

## Introduction

Multitasking, or task switching, is a core competency in emergency medicine [1]. The specialty of emergency medicine requires attention to multiple interruptions as they arise without sacrificing patient care or department flow. Multiple modalities have been considered in the evaluation of emergency medicine physicians with regard to development of the skill of multitasking, including direct observation, task analysis, and simulation education [2,3].

Simulation in particular has been shown to be an effective method of education in emergency medicine, allowing learners to prepare for real-world challenges in a controlled environment [4]. The breadth of simulation education over the years has extended beyond clinical diagnostic scenarios to procedure practice and team management [5]. A study evaluating training in multitasking management of emergency conditions using simulation showed that the vast majority of residents correctly identified and addressed interrupting stimuli and chose safe and correct intervention for their simulated patients [6].

In this study, we evaluated a small cohort of senior residents (postgraduate year [PGY]-2 and 3) in emergency medicine at our institution and their perceived comfort level with a multitasking simulation exercise. Residents were also observed by faculty during the exercise via a two-way glass/mirror, and their performance was used as a guide to assess the residents’ skills for the Accreditation Council for Graduate Medical Education (ACGME) PC-8 (Patient Care-8) milestone, which was designed to assess a resident's skill level in managing complex patients, interruptions, and flow in a busy emergency department (ED) [7]. While the elements in the scoresheet were used to assess learners, this was more of a formative assessment to provide feedback to residents and identify gaps in learning. The scoresheet was used to ensure that critical actions were performed and, if not, to ensure they were discussed in the debrief. Finally, we also tracked residents’ self-assessment of the development of their multitasking skills over time after the majority of senior residents graduated and joined the workforce of attending physicians in emergency medicine. We hypothesized that engagement in this simulation exercise would improve residents’ efficiency in workflow by pointing out areas of both strength and improvement in a safe and controlled environment.

## Materials and methods

Trainees (residents) were given a scenario that simulated the experience of managing two patient encounters within a specified time metric while addressing interruptions that take place in a typical busy ED. The scenario concludes with the resident documenting a chart with an anticipated assignment of an evaluation and management (E/M) level 5. The specific simulation cases and objectives can be found in Tables 1, 2. The following tasks were incorporated into the simulated experience:

1. Assess medical capacity for patients in the prehospital setting.

2. Systematically review electrocardiograms (EKGs) between tasks.

3. Manage patients’ expectations.

4. Task switch in an efficient and timely manner in order to manage multiple patients.

5. Document an ED chart for a level 5 E/M designation.

**Table 1:** Scenario Overview ESP, embedded simulation persons

Case Title	A Simulated Shift to Improve Task Switching in the Emergency Department
Scenario summary	Learners will be on a simulated shift in a single-coverage emergency department and tasked with evaluating two patients, placing appropriate orders, writing both charts, and dispositioning the patients in a timely manner while also managing various interruptions. In this scenario, learners are to prioritize tasks and complete as many tasks as possible in 30 minutes.
Learners	Emergency medicine residents
Learning objectives	To practice efficient task switching while managing multiple patients
	To complete and prioritize necessary tasks in patient care
	To understand the importance of efficiency in a metrics-based emergency department
Required ESP	5 actors (can be played by residents/medical students/standardized patients)
	Patient A
	Patient A’s visitor
	Patient B
	Patient B’s visitor
	Nurse (in person)/paramedic (on a cell phone)
	1 facilitator to complete the assessment
Scenario set-up	Rooms and equipment:
	Room A: one stretcher, one chair
	Patient A in chair, patient A’s visitor sitting on the stretcher
	Room B: one stretcher, one chair
	Patient B in the stretcher, patient B’s visitor sitting in the chair
	Room C: table with computer (for documentation), cell phone (for medical control call)

**Table 2:** Scenario Progression AAO, alert and oriented; BP, blood pressure; CMT, cervical motion tenderness; EKG, electrocardiogram; EMS, emergency medical services; HPI, history of present illness; HR, heart rate; RR, respiratory rate

Anticipated Time	Task	Expected Management
Minute 0-3	Evaluate patient A (older male sitting in a chair with chief complaint of abdominal pain and fever). Visitor sitting on stretcher. The patient will question need for CT scan.	Ask patient A and his visitor to change places to complete an abdominal examination on the stretcher. Discuss need for CT scan (patient will agree after explanation).
Minute 4-7	Evaluate patient B (younger female on the stretcher with a chief complaint of lower abdominal pain, vaginal discharge, and vaginal bleeding). The patient will not make eye contact and will be texting on her phone during the history and physical.	Ask visitor to leave the room to obtain a thorough HPI. Ask patient about sexual history (patient will reveal early pregnancy and history of sexually transmitted infections if the visitor is asked to leave). Perform a pelvic examination (learner will have to verbalize, not actually perform). Pelvic will be reported as closed os, scant bleeding, no CMT or adnexal tenderness. Order labs, ultrasound.
Minute 8-10	Place orders for patients A and B.	Place orders in room C where a computer will be set up and orders will be sent to the facilitator on the program Slack. Order labs, CT scan, pain medicine for patient A. Order labs, ultrasound for patient B.
Minute 11-13	Write level 5 charts for patients A and B.	Document in room C where a computer will be set up with a blank document opened.
Minute 14-15	Respond to an EMS medical control call. EMS calls on a cell phone and provides the following report: “78-year-old female with a history of hypertension, stroke, diabetes, has new onset right-sided weakness and slurred speech. She is refusing transport to the hospital. Her hired caregiver is with her and agrees to watch her at home. Vital signs are BP 201/90, HR 88, O_2_ saturation 95%, RR 18, AAO x 1 (only person).”	Determine capacity of the patient. Since patient is oriented to person only, learner should advise EMS that she is unable to make decisions for herself and must be transported.
Minute 16-18	Continue to document charts.	Document all elements of a level 5 chart until the nurse interrupts with an EKG to be signed.
Minute 19	Sign EKG for a new patient. Nurse will bring a normal EKG to be signed. Nurse will mention that patient A would like to speak to the physician.	Pause documentation to reassess patient A. If resident does not get up to speak to patient A, nurse should continue to prompt learner.
Minute 20	Receive a trauma notification by EMS. Nurse will announce there is a heads up for a trauma alert coming by EMS in 20 minutes. Details of the trauma are not known yet.	The learner may choose to prepare for the incoming trauma, but the trauma patient will not arrive during this scenario.
Minute 21-22	Reassess patient A. Patient A will ask for an update (the learner will not have the results yet) and ask how much longer because he needs to get home to take care of his dog.	Reassess patient’s pain (improved if pain medications were ordered) and answer his questions regarding the wait times.
Minute 23	Sign EKG for a new patient. Nurse will bring an EKG with a prolonged QTc to be signed.	If asked about the new patient’s chief complaint, the nurse should say the EKG is for a patient here for abdominal pain.
Minute 24-26	Complete documentation and review results. Learner will be given the labs and imaging results. The CT read for patient A shows diverticulitis. The ultrasound for patient B shows an intrauterine pregnancy with a reassuring fetal heart rate and a small subchorionic hemorrhage.	The entire chart should be written, including a medical decision-making note for patients A and B.
Minute 27	Respond to nurse’s request for orders. After the nurse notifies the learner of the trauma alert, the nurse will also request a verbal order for ondansetron for the patient who arrived with abdominal pain. (The patient will be the same patient with a prolonged QTc.)	Recognize that the requested ondansetron is for the patient with the prolonged QTc and decline to order it.
Minute 28-29	Discuss the results with patients A and B. The patients will agree with the recommended plan and disposition without any further questioning.	Discuss the results and recommended plan and disposition with patients A and B.
Minute 30	Determine disposition for patients A and B.	Return to room C to write final disposition in a message to the facilitator on the program Slack.

An internally developed scoresheet based on the scenario’s objectives was used to evaluate the resident’s ability to multitask, document a high-level E/M chart, and adhere to the metric of door to disposition (Table 3). The performance of this simulation as a curricular component to improve efficiency was evaluated by an internally developed evaluation survey. The survey was administered using the SurveyMonkey® (San Mateo, CA) platform and consisted of questions eliciting yes/no responses and ratings from a Likert scale. The University of Central Florida Institutional Review Board provided study approval #CR000877.

**Table 3:** Scoresheet EKG, electrocardiogram; EMS, emergency medical services; HPI, history of present illness; MDM, medical decision-making; PE, pelvic examination; PGY, postgraduate year; ROS, review of systems

PGY-3 #1	PGY-3 #2	PGY-3 #3	PGY-3 #4	PGY-2 #1	PGY-2 #2	Task
						Performed history and physical for 2 patients (4 points)
						Entered lab orders for 2 patients (2 points)
						Completed documentation for patient 1:
						HPI with 4 qualifying factors (4 points)
						ROS with 2 in 10 or appropriate verbiage in HPI (10 points)
						PE with 8 organ systems (8 points)
						Appropriate MDM for level 5 chart (5 points)
						Completed documentation for patient 2:
						HPI with 4 qualifying factors (4 points)
						ROS with 2 in 10 or appropriate verbiage in HPI (10 points)
						PE with 8 organ systems (8 points)
						Appropriate MDM for level 5 chart (5 points)
						Responded to EMS radio (2 points)
						Determined incapacity (1 point)
						Signed 2 EKGs (2 points)
						Recognized abnormal EKG (3 points)
						Performed a PE (5 points)
						Managed patient expectations (3 points)
						Total points

## Results

**Figure 1:**
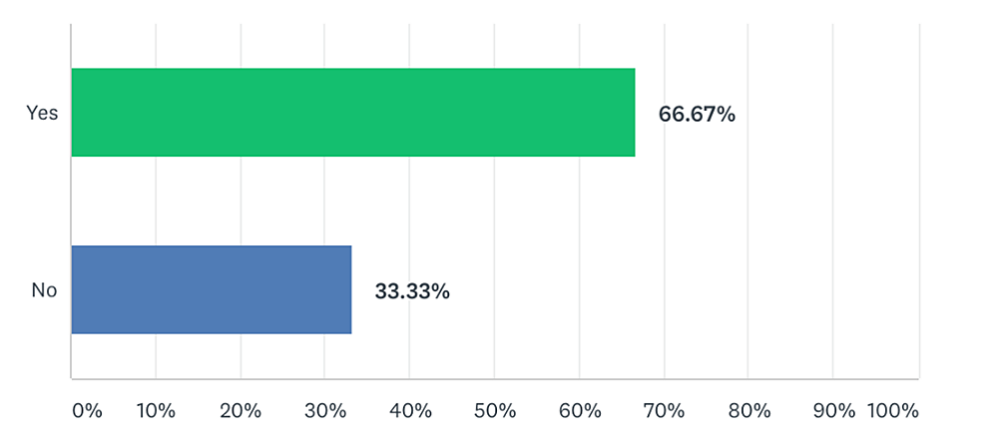
Percentage of residents that reported being specifically trained on task-switching techniques in the past.

**Figure 2:**
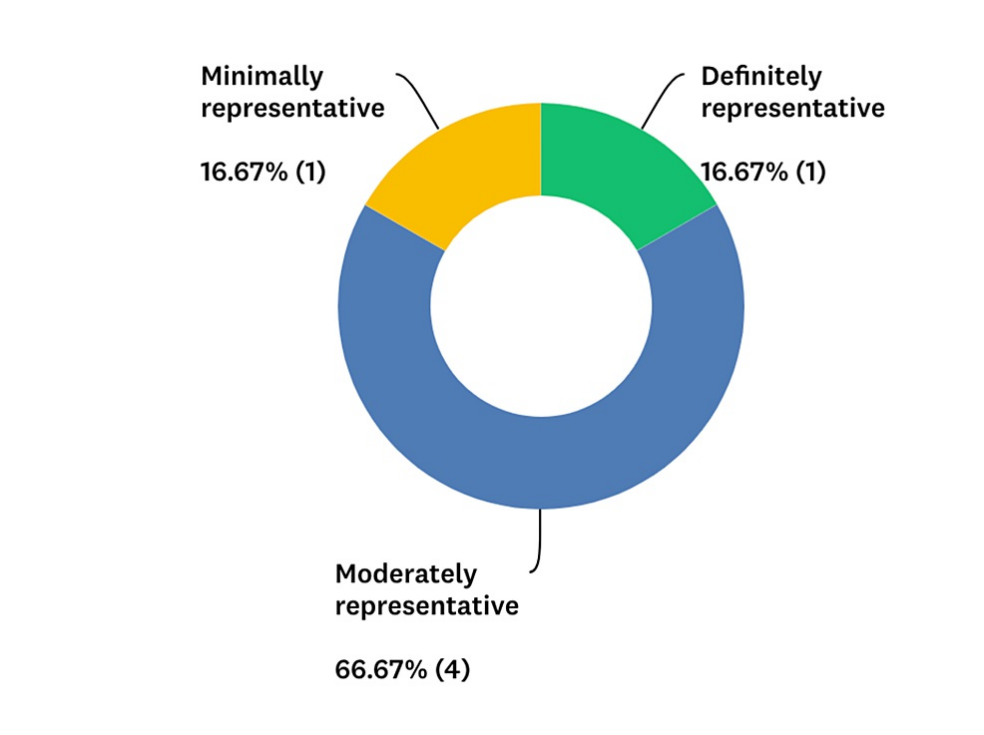
Percentage of residents that felt that this module accurately represented the normal interruptions and flow of our emergency department.

**Figure 3:**
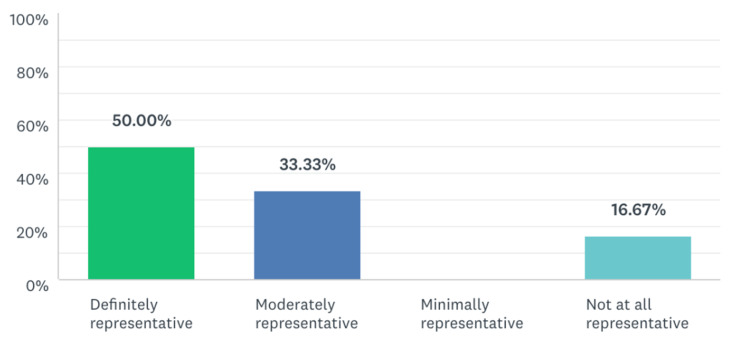
Percentage of residents that felt their performance on this module was representative of their skill level with task switching at the time it was tested.

**Figure 4:**
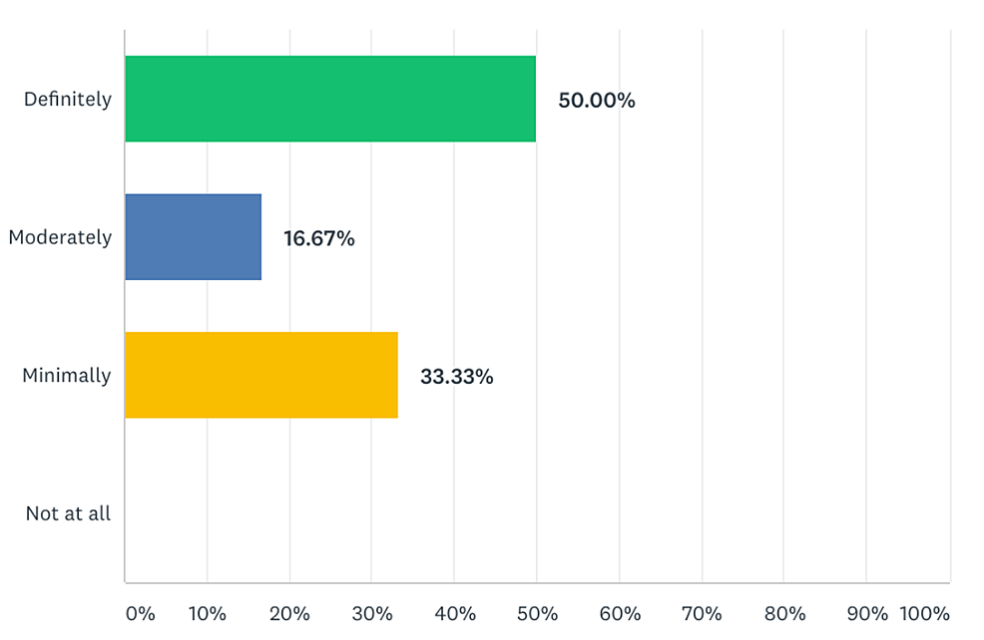
Percentage of residents that felt this module challenged them to practice task switching in a safe space.

**Figure 5:**
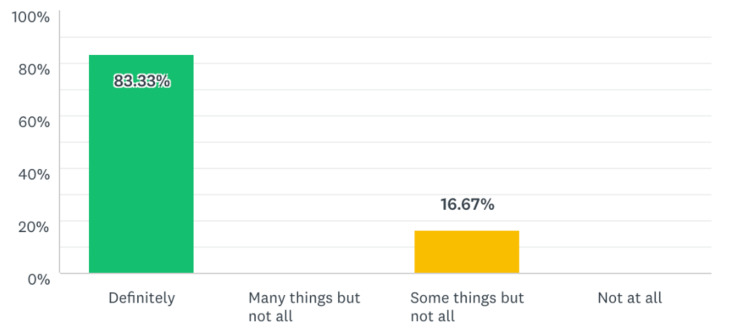
Percentage of residents that will apply skills from this module to their everyday practice.

**Figure 6:**
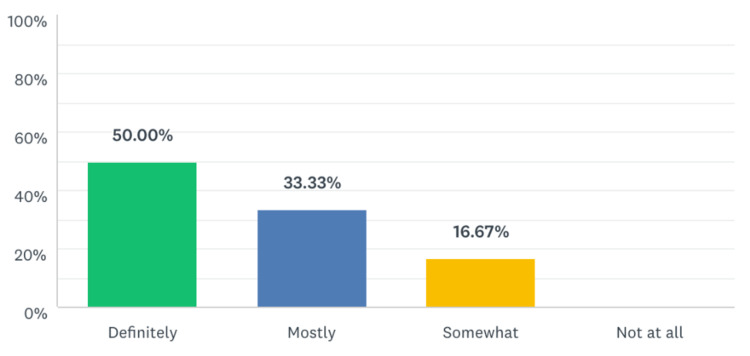
Percentage of residents that have made improvement in their task-switching skills as a result of this module.

**Figure 7:**
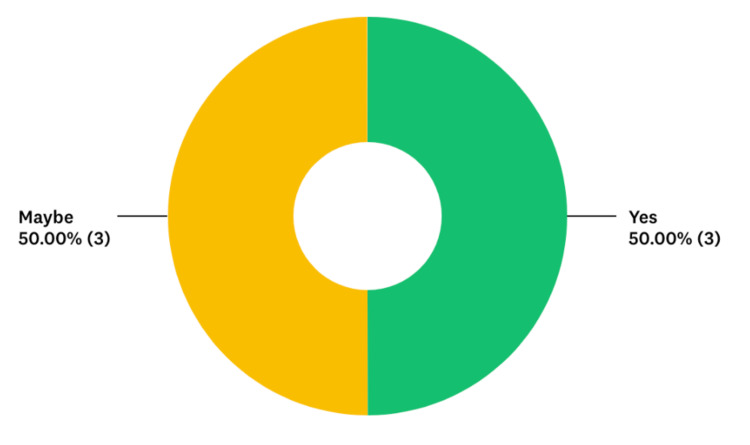
Percentage of residents that would be interested in re-testing their skills with a similar module in the future

The survey response rate was 100%. Figures 1-7 provide the graphical representation of participants’ responses. 

The actual scoresheet for each of the participants is provided in Table 4. All except one resident attended to the patient with the longest wait time first. The remaining resident made this decision based on acuity. Half of the residents brought the computer into the room for entering orders or documenting. Two of the residents utilized the nurse to help with flow. All residents apologized to the patient for any interruptions that occurred during the encounter. All residents were able to at least perform a complete history and physical examination and enter orders for both patients. The following three common themes were pervasive in the debrief discussion: (1) the residents felt the added pressure of the door-to-disposition metric; (2) the objectives of the simulation did not fit within their pre-constructed concept of a successful simulation equating to establishing the correct diagnosis; and, finally, (3) the interruptions were very realistic. Table 5 provides the debriefing guide that was used to highlight teaching pearls in efficiency, documentation, and billing, as well as patient care and communication. It also contains the critical actions that each of the participants were expected to perform.

**Table 4:** Scorecard for Each of the Participants EKG, electrocardiogram; EMS, emergency medical services; HPI, history of present illness; MDM, medical decision-making; PE, pelvic examination; PGY, postgraduate year; ROS, review of systems

PGY-3 #1	PGY-3 #2	PGY-3 #3	PGY-3 #4	PGY-2 #1	PGY-2 #2	Task
4	4	4	4	4	4	Performed history and physical examination for 2 patients (4 points)
2	2	2	2	2	2	Entered lab orders for 2 patients (2 points)
						Completed documentation for patient 1:
3	4	4	4	4	4	HPI with 4 qualifying factors (4 points)
1	10	10	10	10	2	ROS with 2 in 10 or appropriate verbiage in HPI (10 points)
5	8	8	8	8	7	PE with 8 organ systems (8 points)
5	5	5	5	5	5	Appropriate MDM for level 5 chart (5 points)
						Completed documentation for patient 2:
2	2	4	0	4	4	HPI with 4 qualifying factors (4 points)
1	10	10	0	10	5	ROS with 2 in 10 or appropriate verbiage in HPI (10 points)
6	8	8	8	8	7	PE with 8 organ systems (8 points)
3	5	5	0	5	5	Appropriate MDM for level 5 chart (5 points)
2	2	2	2	2	2	Responded to EMS radio (2 points)
1	0	1	1	1	1	Determined incapacity (1 point)
2	2	2	2	2	3	Signed 2 EKGs (2 points)
3	2	2	3	3	2	Recognized abnormal EKG (3 points)
5	0	0	0	0	0	Performed a PE (5 points)
5	3	3	3	3	3	Managed patient expectations (3 points)
50	67	70	52	71	56	Total points

## Discussion

Emergency physicians often work in high-stress environments requiring rapid decision-making with critically ill patients, and this is a key training milestone identified by the ACGME (Task Switching, PC-8) [7]. Emergency physicians are interrupted every 9-14 minutes [8-11], and this number increases with the number of patients managed simultaneously [1]. Task switching and prioritization are key skills in emergency medicine [12-14]. Traditionally, it is “taught” in the clinical setting by emulating senior residents and attending physicians. However, two out of six residents reported that they had not received any specific training in this area. This speaks of the perception that if a skill is taught in the clinical setting, it may not be seen by the learner as a teaching moment.

We therefore developed and instituted a novel teaching technique to train resident emergency physicians on this essential skill. By developing a safe, simulated training environment, we sought to transfer key strategies for improving focus and learning to prioritize while also helping them to identify in themselves how certain pressures and interruptions affected their emotions, stress levels, and concentration. It is important to recognize that multitasking can be stressful. A 2021 study found that ED physicians engaged between 13% and 19% of their work time in multitasking. After controlling for ED workload, they found that physicians who engaged more frequently in multitasking perceived higher work stress [15].

Five out of six residents reported that the simulation experience was moderately or definitively representative of their skill level and the normal interruptions and work flow of our ED. Given that this was a pilot study, we would seek to improve the fidelity of the scenarios with future iterations. All the participants reported that they would translate some of the skills learned in the simulation to their daily clinical practice, and five out of six residents reported improvements in their skills as a result of the task-switching training.

We also included an evaluative component of how fast they were able to reach disposition decisions, perform critical actions, and communicate effectively with their patients. There is increasing attention and emphasis on a number of ED metrics, and although our educational emphasis for junior residents focused on medical management, task prioritization, and patient communication, our senior residents were also expected to address and meet other performance standards related to time to disposition. Our cases concluded with a robust feedback and debrief, as well as a follow-up by email to review critical action competencies.

A number of common themes were noted during the debrief and follow-up. We found that our simulation was very effective in reproducing the real-life pressures to meet disposition times and perform critical actions such as EKG reviews. We also found that our actors playing the roles of nurses and technicians were highly effective in simulating the type and impact of interruptions faced routinely in the ED. Because this was a novel type of simulation, the objectives of the simulation did not fit within the trainees’ pre-constructed concepts and self-identified goals of simulation, which is more typically to perform critical actions and establish the single correct diagnosis and management plan. The emphases instead were on process, focus, prioritization, communication, patient experience, and rapport, elements that became evident to residents soon into each scenario.

As is the case for all simulation-based training, this model allowed us to focus on key thought-and-process concepts in a safe and low-risk environment that allowed trainees to make mistakes and provided opportunity to direct observation and real-time feedback.

There are several limitations to the current study. First, this was a very small pilot study including only six residents and we did not perform a before-and-after analysis to determine the effect of this simulation. Instead, we relied on resident perception, attitude, and self-reported change in behavior following the simulation event. Future studies could examine the effect of this training module on measured clinical efficiency scores. Second, because our simulation center is physically and institutionally separate from the hospital, we were not able to perfectly reproduce the electronic health record, and therefore utilized a low-fidelity system for order entry and documentation, which was not consistent with their true practice. Future simulation training will better reproduce the true practice environment. Finally, while the focus for documentation was to be thorough and complete in order to create a level 5 chart, the specific patient encounters may not be assigned an E/M level 5 and this may have led to unnecessary time spent documenting given the particular case. Future studies should align the case acuity with the documentation and billing level in order to provide greater realism and create additional opportunities for learner improvement.

## Conclusions

Using simulation, residents were evaluated on the core competency of multitasking. Residents were surveyed regarding whether or not it accurately simulated a real-world scenario and if they learned skills that would translate into their clinical practice. We found that residents enjoyed this activity and felt that they obtained skills that they would implement in their every day. In the future, this activity can be repeated with equipment similar to that found in the hospital and with a greater number of residents.

## Appendices

**Table 5:** Debriefing Guidelines AAO, alert and oriented; BP, blood pressure; CMT, cervical motion tenderness; CVA, cerebrovascular accident; DM, diabetes mellitus; ED, emergency department; EKG, electrocardiogram; E/M, evaluation and management; EMS, emergency medical services; HPI, history of present illness; HR, heart rate; HTN, hypertension; MDM, medical decision-making; PE, pelvic examination; PMH, past medical history; PFSH, past, family, social history; ROS, review of systems; STI, sexually transmitted infection

	Debriefing Guidelines	
Debrief General Outline		
	Summary and Objectives	
	“This simulation was about task switching in the emergency department. The objectives of this scenario include management of multiple patients, prioritization of tasks, and understanding the importance of efficiency in a metrics-driven ED.”	
	Reactions	
	"How do you feel?” or “Any initial thoughts on how that went?"	
	“What went well and what could have been done differently?”	
	Analysis	
		Try to understand the learner’s perspective on task switching and allow for self-reflection.
		Provide feedback on positive performance and areas for improvement.
		Deliver teaching points (refer to teaching points below).
	Summary	
		“After participating in this simulation, what will you incorporate into your practice?”
		“Any other personal takeaway points?” (reinforce teaching points here)
Debrief: Teaching Pearls		
	Efficiency	
		Perform the history and physical examination for both patients prior to sitting down to document.
		Consider taking a computer into the patient’s room to document and enter orders while evaluating the patient.
		Perform procedures early. Once the need for a procedure is identified, bring necessary supplies into the room.
		Determine the most likely disposition for each patient immediately after completing the initial history and physical examination.
		Write and review the medical decision-making section as you are entering orders to ensure all appropriate orders are placed and to avoid late entry of orders.
	Documentation/Billing	
		“4-2-1” rule: four descriptors for HPI, two sections of PFSH, and one item per system for 10 ROS guarantees a comprehensive history (1).
		Create a chart template to routinely document all charts at an E/M level 5 when appropriate.
		Utilize dictation software when available.
	Patient Care and Communication	
		Discuss treatment plan with patient and ask if there are any questions after the initial assessment.
		Preemptively acknowledge any potential time delays before leaving the patient’s room.
		Be systematic when performing history and physical examination and when reviewing results such as labs, imaging, and EKGs to ensure key findings do not go unnoticed.
		Focus on patient communication best practices: introducing self and team, sitting down at the bedside, setting expectations.
Simulation Case		
		The most senior resident will compete against time and other residents to see who can complete the most tasks in 30 minutes. You are single coverage in a very busy ER. You have two nurses. Administration is very sensitive to your metrics. There is a huge emphasis on door to disposition. Time:door starts once you enter the room. Time:disposition is when the chart is completed in a manner that anticipates an E/M level 5. Your job is on the line if your metrics are not sufficient.
		Tasks to give to resident: (1) complete two HPIs and PEs; (2) enter lab orders (on Slack) for both patients; (3) document two level 5 charts with a good MDM; (4) establish a disposition within the specified time frame (30 minutes) (door-to-disposition time < 30 minutes).
	Actors	
		two patients (played by residents), one friend in patient A’s bed and one friend in patient B’s room (boyfriend), and nurse with the 2 EKGs, who also acts as a helper paramedic who calls the resident during shift.
	Set-Up/Materials	
		two stretchers in one room (patient A in chair, patient B in bed already), two chairs, small table with computer for documenting, phone for medical control call, and 2 EKGs, (one with prolonged QTC).
	Critical Actions	
		Complete two HPIs
		Ask Patient A to switch places with his friend for an appropriate abdominal examination.
		Ask patient B’s boyfriend to step out of the room to get a full HPI.
		Enter lab orders (on Slack) for both patients.
		Document two level 5 charts with a good MDM.
		Respond to medical control call.
		Determine EMS patient to be incapacitated and not able to make decisions for herself.
		Hidden task: perform a pelvic examination (don’t tell the resident).
		Sign two EKGs.
		Avoid giving ondansetron to the patient with the abnormal EKG.
		Provide a disposition for both patients.
	Cases	
		Patient A: older male sitting in a chair, next to the hospital bed (friend is relaxing in his bed. Chief complaint: abdominal pain, fever. Patient questions need for CT. Eventually agrees for CT. Requires pain medication. Outcome: diverticulitis requiring admission.
		Patient B: younger female (not much eye contact, texting on her phone). Chief complaint: lower abdominal pain/vaginal discharge/vaginal bleeding. Will require a pelvic examination; boyfriend is in the room until asked to step out by physician. Diagnosis: STI, pregnancy, subchorionic hemorrhage. Disposition: discharge home.
		Trauma alert heads up announced at 20 minutes in to case (trauma alert doesn’t actually arrive during the case).
	Instructions for Interruptions	
		Quick EMS radio call: 78-year-old female; PMH: HTN, CVA, DM, has right-sided weakness and slurred speech. Doesn’t want to go to the hospital. She is at the house with a hired caregiver who says she doesn’t mind watching her. Vitals: BP 201/90, HR 88, O_2_ 95%, 18; AAO x 1 (person, not place, not time).
		Sign 2 triage EKGs brought in intermittently by nurse.
		Nurse interrupts resident to inform them of patient’s request to speak with them again (if asked why, the nurse should say, I’m not sure).
		Patient (resident actor) asks a quick question like: “do you know how long I’m going to be here, I may need to arrange care for my dog.”
		Nurse comes to ask physician for a verbal order for Zofran for a patient in triage, it’s the one whose EKG was signed (hint: QTc 512; don’t verbalize to the resident).
Debriefing and Evaluation Pearls (10 minutes)		
	Patient Care and Communication Pearls	
		Be systematic about EKGs so that key findings do not go unnoticed.
		Become familiar with local EMS protocols, especially involving medical incapacitation. Assess for the patient’s ability to make an informed decision.
		Avoid asking extraneous information from EMS, recognizing care is time-sensitive, often involves distraught family members, and is resource-poor relative to the ED.
		Prior to leaving the patient’s room, ask if they have any questions and preemptively acknowledge any potential time delays, while informing them of their treatment plan.
	Pearls for Efficiency	
		It’s helpful to perform the history and physical of both patients prior to sitting down to document.
		Take the computer in the room and document/enter orders while in the patient’s room
		Do procedures early. Once the chief complaint identifies the need for a procedure, bring the supplies into the room in anticipation.
		Determine the most likely disposition for the patient once the history and physical is performed.
		Review the medical decision-making section at the same time orders are being entered, as to avoid late entry of orders.
	Pearls for Documentation/Billing	
		“4-2-1” rule: four descriptors for HPI, two sections of PFSH, and one item per system for 10 ROS guarantees a comprehensive history (1).
		Create a chart template to routinely document all charts at an E/M level 5 when appropriate.
		Utilize dictation software for the HPI when available.
	Suggested Debriefing Questions	
		What did you feel was difficult or challenging about the medical control call?
		What are some strategies you can employ to help with task switching?
		What systematic process could you employ to help with timely and accurate interpretation of EKGs?
		What verbiage can you use to help manage patients’ expectations in a busy ED?
		What are some additional distractions that affect your ED workflow?
